# Asymptotic dispersion engineering for ultra-broadband meta-optics

**DOI:** 10.1038/s41467-023-42268-5

**Published:** 2023-10-20

**Authors:** Yueqiang Hu, Yuting Jiang, Yi Zhang, Xing Yang, Xiangnian Ou, Ling Li, Xianghong Kong, Xingsi Liu, Cheng-Wei Qiu, Huigao Duan

**Affiliations:** 1 National Research Center for High-Efficiency Grinding, College of Mechanical and Vehicle Engineering, Hunan University, Changsha, 410082 PR China; 2 Advanced Manufacturing Laboratory of Micro-Nano Optical Devices, Shenzhen Research Institute, Hunan University, Shenzhen, 518000 PR China; 3 Department of Electrical and Computer Engineering, National University of Singapore, Singapore, Singapore; 4 Greater Bay Area Institute for Innovation, Hunan University, Guangzhou, 511300 PR China

**Keywords:** Metamaterials, Nanophotonics and plasmonics

## Abstract

Dispersion decomposes compound light into its monochromatic components, which is detrimental to broadband imaging but advantageous for spectroscopic applications. Metasurfaces provide a unique path to modulate the dispersion by adjusting structural parameters on a two-dimensional plane. However, conventional linear phase compensation does not adequately match the meta-unit’s dispersion characteristics with required complex dispersion, hindering at-will dispersion engineering over a very wide bandwidth particularly. Here, we propose an asymptotic phase compensation strategy for ultra-broadband dispersion-controlled metalenses. Metasurfaces with extraordinarily high aspect ratio nanostructures have been fabricated for arbitrary dispersion control in ultra-broad bandwidth, and we experimentally demonstrate the single-layer achromatic metalenses in the visible to infrared spectrum (400 nm~1000 nm, NA = 0.164). Our proposed scheme provides a comprehensive theoretical framework for single-layer meta-optics, allowing for arbitrary dispersion manipulation without bandwidth restrictions. This development is expected to have significant applications in ultra-broadband imaging and chromatography detection, among others.

## Abstract (web summary)

The authors present a comprehensive framework for on-demand dispersion control with a single-layer metasurface, particularly in an ultra-broad bandwidth. An achromatic metalens spanning the visible and near-infrared spectra is experimentally demonstrated.

## Introduction

Dispersion is a fundamental property of materials, i.e., the refractive index of material with normal dispersion (e.g., glass) decreases with increasing wavelength. As a result, the prism deflects light at a longer wavelength by a smaller angle, and the focal lengths at a longer wavelength of refractive lenses are larger than for a shorter wavelength (i.e., positive chromatic aberration). The opposite is true for dispersion in diffractive optical elements. The manipulation of dispersion has been of wide interest and has numerous important applications. For example, the chromatic aberration in lens imaging^1^ and near-eye displays^2^ severely degrades the image quality, thus chromatic aberration should be eliminated. Conversely, enlarging dispersion can enable higher resolution of spectrometer devices^3^ and transmission capacity of wavelength division multiplexing in optical communications^4^. Even arbitrary modulation of dispersion is required in applications such as color holographic displays^5,6^, color router^7,8^, and computational spectral imaging^9,10^. Furthermore, optical elements always require longer operating bands to ensure seamless operation across each band without the need for component replacement. However, the manipulation of dispersion usually requires the stacking of multiple components leading to bulky and complex systems, which are not conducive to the applications of miniaturized optical systems in consumer electronics, wearable devices, and miniature spectrometers.

Metasurfaces provide an attractive platform to design ultra-thin planar optical elements by constructing subwavelength scatters (meta-units) in a two-dimensional plane^11–13^. Combined with the multiparametric control capability of meta-units, a variety of elements with plentiful functionality, such as beam deflectors, metalenses^14–17^, metaholograms^18–21^ and complex beam generators^22–24^, have been demonstrated. However, most of these elements have fixed severe negative chromatic aberrations, similar to those of conventional diffractive elements. Previous efforts have been done to achieve dispersion manipulation, mainly chromatic aberration elimination, at discrete wavelengths or broadband through spatial multiplexing, such as sub-region^25–27^, interleaved meta-units^28–30^ and stacking layers^31–35^. Meanwhile, with structural dispersion design freedom of different subwavelength structures, the chromatic aberration elimination at discrete wavelengths^36^, narrowband^37,38^, and broadband^39–46^ in different wavelength bands with a single-layer non-interleaved metalens can be realized through linear phase compensation. However, this scheme is challenging for designing arbitrary dispersion-controlled meta-optics, especially in ultra-broadband cases. Linear phase compensation is capable of achieving approximate fitting for monotonic dispersion engineering (positive dispersion, negative dispersion, no dispersion) within a certain bandwidth^37,45^. However, when the bandwidth continuously increases, the intrinsic nonlinear dispersion of the structure becomes mismatched with the linear phase dispersion, resulting in a significant increase in wavefront error. This can cause degradation in efficiency or resolution, and can even prevent the achievement of the intended design function. For non-monotonic dispersion engineering, linear compensation results in an even more severe mismatch and fails to achieve the intended arbitrary dispersion design, even in a narrow bandwidth. Meanwhile, the limited height of subwavelength structures used in previous experiments also poses difficulties in covering a large phase compensation range, which hinders the realization of large bandwidths while maintaining the sizes and NA.

In this study, we present a generalized asymptotic phase compensation strategy for the development of at-will dispersion-controlled meta-optics, especially for an ultra-broad bandwidth. The proposed method allows for the arbitrary customization of the design wavefront under different wavelengths through translation, achieving an asymptotic match with the phase dispersion of the meta-units. This approach offers a pathway to expand the operation bandwidth of the dispersion-controlled elements. Through the use of high-aspect-ratio TiO_2_ nanostructures with a height of 1000 nm and a minimum width of 50 nm, we experimentally demonstrate the ultra-broadband single-layer achromatic metalenses across the visible to infrared range (400~1000 nm, NA = 0.164). When combined with a mature CMOS image sensor, this metalens demonstrates the ability to provide 24-h imaging detection, with applications in security surveillance and consumer electronics. Our approach is further utilized to realize customized dispersion (monotonic and non-monotonic dispersion along the optical axis and wavelength routing on the focal plane). Overall, this method improves the theoretical framework of single-layer achromatic metalens design and offers a general method to achieve more accurate phase matching and greater degrees of freedom for dispersion modulation.

## Results

**Fig. 1:**
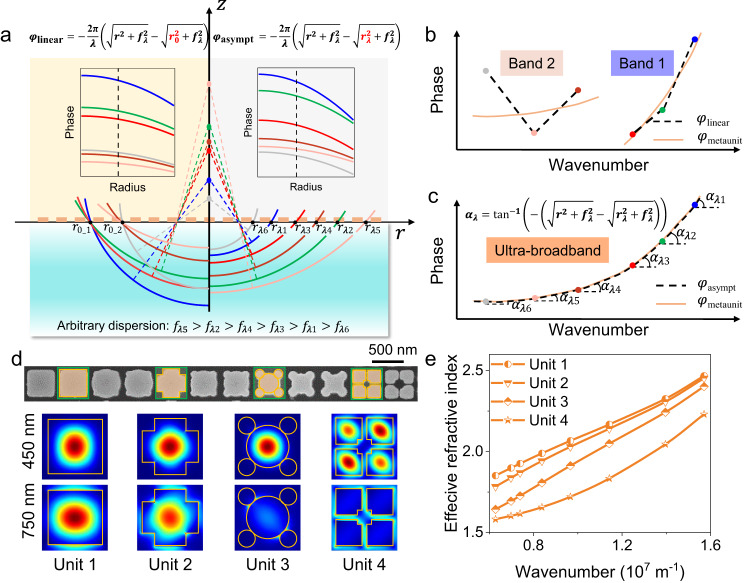
The schematic of dielectric metalenses for at-will dispersion engineering in an ultra-broad bandwidth. **a** The schematic wavefronts of two different compensation methods: linear phase compensation and asymptotic phase compensation, for arbitrary dispersion manipulation. The left and the right embedded figure are the phase profiles of different wavelengths of the two methods, respectively. The wavelength decreases from gray to blue. **b** The comparison between the intrinsic phase dispersion of the meta-unit and constructed phase dispersion of linear phase compensation method in two narrow bands. **c** The comparison between the intrinsic phase dispersion of the meta-unit and constructed phase dispersion of asymptotic phase compensation method in the ultra-broadband range. **d** Scanning electron microscope image of the four types polarization-insensitive meta-units and the waveguide modes at two different wavelengths. **e** The relationship between the effective refractive index of four meta-units and the wavenumber.

Achieving arbitrary manipulation of the dispersion of an optical element requires the design of independent phase profiles at all designed wavelengths. For a lens, it is necessary to focus light of different wavelengths to their respective focal points as shown in Fig. 1a. This requires designing wavelength-dependent phase profiles at the interface of the lens achieved by the meta-units to construct a hemisphere wavefront with the designed spherical center. The wavefront constructive interference of each light path from the interface should accumulate the propagating phase in free space, represented by the equation $\varphi=-{k}_{\lambda }d=-\frac{2{{{{{\rm{\pi }}}}}}}{\lambda }d$, where ${d}$ is the physical distance between the wavefront and the interface at a specific location (see Fig. S1). Therefore, the construction of $d$ determines the phase relationship between different wavelengths, i.e., phase dispersion, which should align with the structural dispersion of the meta-units to achieve the required phase compensation at each wavelength. The phase profile of the metalens in previous studies using linear phase compensation methods^40,46,47^ is commonly constructed as ${\varphi }_{{{{{\rm{linear}}}}}}(r,\lambda )=-\frac{2{{{{{\rm{\pi }}}}}}}{\lambda }(\sqrt{{r}^{2}+{f}_{\lambda }^{2}}-\sqrt{{r}_{0}^{2}+{f}_{\lambda }^{2}})$, where ${f}_{\lambda }$ is the focal length for each wavelength and ${r}_{0}$ represents the intersection point of the wavefront plane with the metalens surface. In achromatic lens designs, where ${f}_{\lambda }$ is a constant value, a fixed ${r}_{0}$ results in the constructed phase ${\varphi }_{{{{{\rm{linear}}}}}}$ being positively linear with wavenumber for positions smaller than ${r}_{0}$. This approach can achieve an approximate linear fit with the meta-units’ structural dispersion, which is also positively correlated with the wavenumber but nonlinear, and thus eliminate chromatic aberration within a certain bandwidth (see Fig. S1). However, the left side of Fig. 1a and Fig. 1b illustrate the challenge of designing at-will dispersion modulation, i.e., a variable focal length ${f}_{\lambda }$, using linear compensation, especially in ultra-broadband. In the case of smaller variations in focal length design in band 1 (${\lambda }_{1}$ < ${\lambda }_{2}$ < ${\lambda }_{3}$, ${f}_{{\lambda }_{1}}$ < ${f}_{{\lambda }_{3}}$ < ${f}_{{\lambda }_{2}}$), a fixed ${r}_{0{{{{{\rm{\_}}}}}}1}$ is chosen, and its required phase dispersion still shows a monotonically positive correlation with the wavenumber, enabling an approximate fit with the structural dispersion with some error, as shown in Fig. 1b. Nevertheless, for large variations in focal length in band 2 (${\lambda }_{4}$ < ${\lambda }_{5}$ < ${\lambda }_{6}$, ${f}_{{\lambda }_{6}}$ < ${f}_{{\lambda }_{4}}$ < ${f}_{{\lambda }_{5}}$), the demanded phase dispersion appears non-monotonic due to the fixed ${r}_{0{{{{{\rm{\_}}}}}}2}$, and it becomes more challenging to achieve an approximate fit with the structural dispersion to achieve the intended design, as shown Fig. 1b. If one wishes to achieve customized dispersion control over an even broader bandwidth, such as band 1+band 2, it becomes almost impossible to accomplish using the linear phase compensation method.

To achieve arbitrary dispersion customization in ultra-broadband and better match with the nonlinear structural dispersion, we propose an asymptotic phase compensation approach for phase dispersion construction, as shown on the right side of Fig. 1a and expressed as

1 $${\varphi }_{{{{{\rm{asympt}}}}}}(r,\lambda )=-\frac{2\pi }{\lambda }\left(\sqrt{{r}^{2}+{f}_{\lambda }^{2}}-\sqrt{{r}_{\lambda }^{2}+{f}_{\lambda }^{2}}\right)$$

This approach involves introducing a wavelength-dependent value, ${r}_{\lambda }$, which allows for the arbitrary translation of the wavefront plane for each wavelength to construct phase dispersion at will. As a result, the local phase relationship can be arbitrary constructed to asymptotically match the nonlinear structural phase dispersion, enabling high-fidelity arbitrary dispersion engineering in the ultra-broadband range as shown in Fig. 1c. In the design process, the wavelength-dependent phase ${\varphi }_{{{{{\rm{asympt}}}}}}(r,\lambda )$ required for each position can be decomposed from Eq. (1) into two parts, ${\varphi }_{{{{{\rm{asympt}}}}}}\left(r,\lambda \right)=\varphi \left(r,{\lambda }_{\max }\right)+\Delta \varphi \left(r,\lambda \right)$, where the first term is the phase at the maximum wavelength of the achromatic band (i.e., the base phase), and the second term is the phase difference between the wavelength of interest $\lambda$ and ${\lambda }_{\max }$, reflecting the phase dispersion. Therefore, to design ultra-broadband dispersion-controlled metalenses, appropriate ${r}_{\lambda }$ values should be constructed and appropriate meta-unit at each position should be selected to satisfy the required phase and phase dispersion.

The phase dispersion of subwavelength structures, namely meta-units, is strongly correlated with their cross-sectional shapes, offering new opportunities for dispersion manipulation with metasurfaces. To comprehend the structural dispersion mechanism of dielectric subwavelength meta-units, we view the meta-units as vertical waveguides, where their phase response can be expressed as ref. ^11^

2 $${\varphi }_{{{{{\rm{meta}}}}}}={\frac{2\pi }{\lambda }n_{{{{{\rm{eff}}}}}}}(\lambda )H$$

where $H$ is the height of the meta-unit. ${n}_{{{{{\rm{eff}}}}}}(\lambda )$ is the effective refractive index (ERI) related to the intrinsic refractive index and cross-section shape of the meta-unit. Based on Eq. (1), for a fixed height meta-unit, the phase is positively correlated with the wavenumber, and the exact relationship is determined by the ERI. In previous studies, the phase dispersion of the meta-unit was assumed to be linear, i.e., the variation of the ERI with wavelength was ignored in order to compensate for the constructed linear phase dispersion of the metalens. However, based on the one-dimensional equivalent medium theory, the ERI of a material usually changes with the wavelength of light (see Section 2 in the Supplementary Information). In the case of two-dimensional meta-units, which have more complex cross-sectional shapes, we modeled them as dielectric waveguides in transmission mode, with wavelength-dependent ERI that were calculated using eigenmode analysis (as described in the “Methods” section). TiO_2_ material is chosen to achieve the visible to near-IR design due to its optical constant measurement result in Fig. S3, which shows a large refractive index with negligible absorption in this band. Figure 1d shows four types of polarization-insensitive meta-units, each with specific dimensional parameters and corresponding waveguide modes at two different wavelengths. These modes exhibit significant differences across wavelengths, resulting in different ERIs at different wavelengths, as shown in Fig. 1e. Therefore, we can conclude from Eq. (2) that the meta-units have nonlinear phase dispersions due to the varying ERIs with respect to wavelength (as illustrated in Fig. S4) and this effect will be more pronounced when the bandwidth increases. Therefore, the proposed asymptotic phase compensation can perfectly match the phase dispersion property of the meta-unit.

**Fig. 2:**
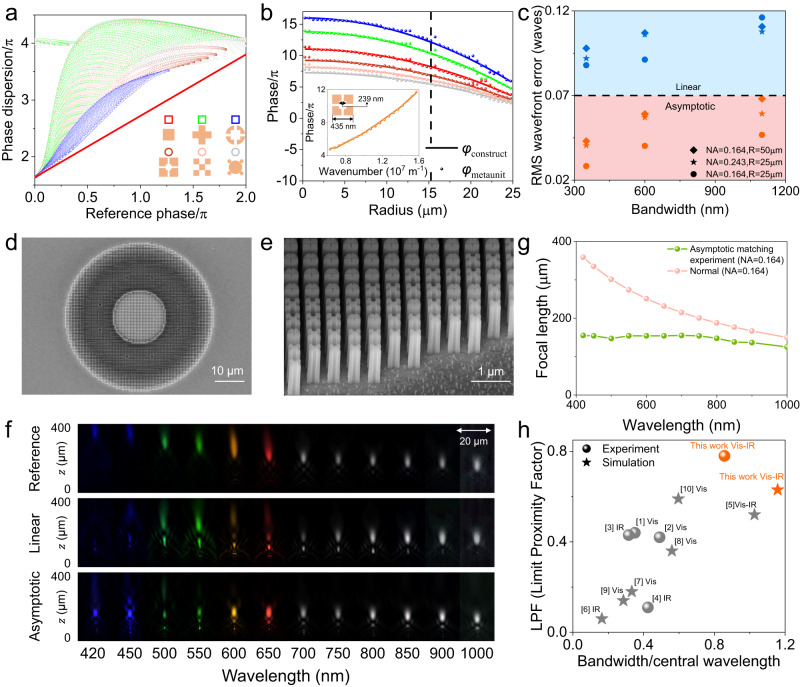
The design process and experimental results of ultra-broadband achromatic dielectric metalenses. **a** The “phase-phase dispersion” library of meta-units calculated at 550 nm wavelength, where ${{{{{{\rm{\lambda }}}}}}}_{\max }$ = 1000 nm. The red line is the effective medium line representing the theoretical limit with minimal dispersion (no structural dispersion). **b** The asymptotic matching results in the radius dimension for a metalens with a radius of 25 μm and NA of 0.164 at different wavelengths, where the solid lines represent the constructed wavefront phase profiles and the scatters denote the phases of the matched meta-units. The embedding image is the phase dispersion figure of the structure at the position of 15.25 μm radius. **c** The RMS wavefront error of the metalenses with different NAs. The blue scatters and the orange scatters are the linear matched group and the asymptotic matched group, respectively. The NAs and lens sizes of the circular scatters, the star scatters and the rhombic scatters are NA = 0.243, *R* = 25 μm, NA = 0.164, *R* = 25 μm, and NA = 0.164, *R* = 50 μm respectively. **d**, **e** Scanning electron microscope (SEM) images of the fabricated achromatic metalens. **f** The experimental intensity distribution along the propagation direction (z axis) of the three metalenses. From top to bottom are the experimental results of the reference group, the linear matched group and the asymptotic matched group, respectively. **g** Focal length distribution at different wavelengths of metalens with NA = 0.164. The green line is the focal length of the asymptotic matched metalens, and the pink line is the normal negative dispersion reference curve. **h** The comparison of the performance of the single-layer achromatic metalens achieved in our work with previous studies in terms of bandwidth and limit proximity factor dimensions. The circular scatters and the star scatters are the experimental and simulation results, respectively. The working bands of the gray scatters are visible and near infrared, the orange scatters are from visible to near infrared.

To achieve the desired asymptotic phase compensation at different locations of the metalens, a large library of polarization-insensitive dielectric meta-units with six different cross-sectional shapes (including the four shapes depicted in Fig. 1d) and dimensional parameters was constructed. Since the reference phase (phase at *λ*_max_) and phase dispersion are the key parameters of the meta-units, library of meta-units with 1000 nm height and various shapes or sizes are plotted in the “phase-phase dispersion” space as shown in Fig. 2a for a wavelength of 550 nm (with a maximum wavelength of 1000 nm). The phase dispersion results for other wavelengths and 600-nm-height structures are presented in Section 5 of the Supplementary Information, demonstrating that higher structures can compensate for a greater range of dispersion. Each point in the plot represents a meta-unit with a particular parameter under a specific shape. The subclasses of each shape occupy distinct regions in the plot, indicating that they exhibit different levels of structural dispersion. Our library densely covers almost all possible structural dispersion regions, as demonstrated by the effective medium line representing the theoretical limit with minimal dispersion (i.e., no structural dispersion), thereby ensuring appropriate dispersion meta-units can be found at different locations. The simulated transmittance results of the six meta-units and the metalens throughout the visible to near-infrared in Figs. S8 and S9 also show a good transmission performance.

Using the established library of meta-units, we first designed the most typical dispersion-modulated meta-optics, achromatic metalenses, with different bandwidths (350, 600, 1100 nm from wavelength of 400 nm), different lens size (up to 100 μm) and different NAs (up to 0.243). We utilized both linear and asymptotic phase compensation approaches for comparison. The particle swarm optimization (PSO) algorithm is employed in the asymptotic phase compensation approach to optimize the value of ${r}_{\lambda }$ at each wavelength, with the aim of minimizing the matching errors between the required phase dispersion and that of the meta-units, as described in the Methods section. Figure 2b shows the asymptotic matching results for a metalens with a radius of 25 μm and NA of 0.164 at different wavelengths across 400–1000 nm, where the solid lines represent the constructed wavefront phase profiles and the scatters denote the phases of the matched meta-units (see Section 7 in the Supplementary Information for other matching results). The intrinsic phase dispersion of the selected meta-unit matches better with the constructed asymptotic phase dispersion (embedding plots) in the large operation band, making the asymptotic phase compensation approach superior to the linear ones in most wavelengths. To further highlight the advantage of the asymptotic phase compensation approach over the linear ones, we calculated the average values of root mean squares of the wave aberration function (${{{{{\rm{WAF}}}}}}_{{{{{\rm{rms}}}}}}$= $\sqrt{{\langle {{{{{\rm{WAF}}}}}}\rangle }^{2}-\langle {{{{{{\rm{WAF}}}}}}}^{2}\rangle }$, where the brackets represent the mean value) for metalenses with different bandwidths, NAs and lens sizes, as shown in Fig. 2c. According to the Marèchal criterion, aberrations are negligible when ${{{{{\rm{WAF}}}}}}_{{{{{\rm{rms}}}}}}$/λ< 0.071, implying that metalenses exhibit theoretical diffraction-limited focusing performance. We observed that the asymptotic compensation approach achieves ${{{{{\rm{WAF}}}}}}_{{{{{\rm{rms}}}}}}$ values less than 0.071λ for different lens sizes and NAs as the bandwidth increases, while the opposite holds true for linear compensation method. Due to the fact that the dispersion compensation range is approaching its limit, the error increases as the bandwidth increases. The MATLAB simulated intensity distribution along the propagation direction in Figs. S11–S13 provides more intuitive evidence that the linear compensation approach exhibits a multi-focal phenomenon and a large shift in focal points as the bandwidth increases, suggesting that the wavefront error is too large to achieve the intended function. In contrast, the asymptotic compensation approach consistently delivers better achromatic performance.

To achieve high-quality preparation of metalenses consisting of high aspect ratio meta-units, a conformal filling approach based on electron-beam lithography (EBL) and atomic layer deposition (ALD) was implemented, as described detailed in the “Methods” section and Fig. S14^16,48^. The processed structures had an aspect ratio of 20, with a height of 1000 nm and a minimum size of 50 nm, enabling a larger dispersion range to be covered. Achromatic metalenses with NA of 0.164 and 50 μm diameter were prepared. SEM images of the fabricated metalens are shown in Fig. 2d, e, which demonstrate that the fabrication process effectively maintained the steepness of the nanostructures. Experimental characterization of the fabricated metalenses was conducted using the optical setup described in Section 10 of Supplementary information, which involved measuring the focusing and imaging performance. Figure 2f shows the experimental intensity distribution along the propagation direction (*z* axis) of the three metalenses with NA = 0.164. The reference group exhibited normal negative dispersion characteristics of a single-wavelength designed metalens, with the focal length decreasing as the wavelength increased. The linear matching metalens achieved achromatic focusing in a certain bandwidth (e.g., 750–900 nm) but had poor focusing in the visible range due to large matching errors. The metalens designed with asymptotic phase compensation, however, achieved preferable chromatic aberration elimination from 400 to 1000 nm. Experimental results were measured from the 420 nm wavelength due to the limit of the minimum wavelength of the light source (see Fig. S12 for simulation results of the 400 nm wavelength). Focal length of the asymptotic matching metalens are shown in Fig. 2g, which demonstrates that the designed metalens achieved achromatic focusing in the operation band. Based on the efficiency performance compared with other works shown in Fig. S17, the proposed metalens can achieve good efficiency performance in ultra-broadband. However, there is still some efficiency loss due to processing errors. Also, the observed shift in focus during the experiment can be attributed to the inaccuracies of the fabrication process^49–51^. Further analysis of the effects of process inaccuracies is provided in Section 12 of the Supplementary Information.

Single-layer achromatic metalenses designed using phase compensation methods are limited by the maximum phase dispersion (i.e. group delay) that the structure can provide^46,52,53^. Therefore, a single metric such as bandwidth cannot fully evaluate the design. To address this, we have introduced a more comprehensive evaluation factor, *χ* (limit proximity factor, LPF), which is based on the maximum phase dispersion limit^46^. The LPF is calculated using

3 $$\chi=\frac{R\cdot {{{{{\rm{NA}}}}}}\cdot {{{{{\rm{\pi }}}}}}\left(\frac{1}{{\lambda }_{\min }}-\frac{1}{{\lambda }_{\max }}\right)}{{\Delta \varphi }_{\max }}$$

where $R$, $\,{{{{{\rm{NA}}}}}}$, (${\lambda }_{\max }$, ${\lambda }_{\min }$) denote the radius, NA, and operating bandwidth of the achromatic metalens. ${\Delta \varphi }_{\max }$ is the maximum phase dispersion that the structure can provide. Thus, the LPF expresses the comprehensive performance achieved under the maximum phase dispersion, accounting for lens size, NA, and bandwidth. The closer the ${{{{{\rm{LPF}}}}}}$ is to 1, the closer the design is to the limit. Figure 2h presents a comparison of the performance of the single-layer achromatic metalens achieved in our work with previous studies in terms of bandwidth and LPF dimensions, using both experimental and simulation results. In our study, we have accomplished the broadest bandwidth among current experimental investigations, while obtaining the highest LPF, indicating that the achieved bandwidth was not compromised by metalens size or NA. It is also the first experimental realization of a single-layer achromatic metalens from the visible to the near infrared. Additionally, through simulations, we have designed achromatic metalenses with larger NA or broader bandwidth (400~1500 nm), representing the highest level of simulation metrics available. Detailed comparative data are provided in Supplementary Information Table S1.

**Fig. 3:**
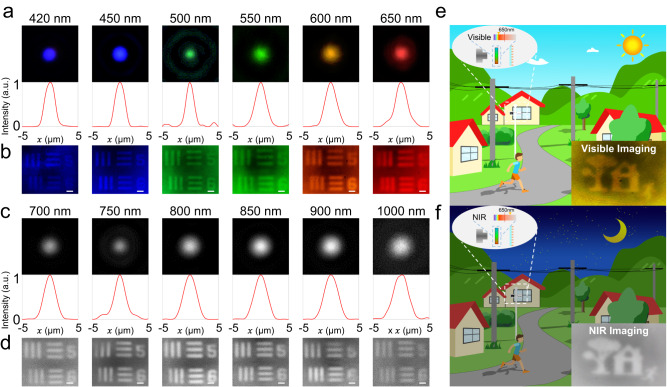
The focus characterization and imaging performance of the asymptotic matching achromatic metalens with NA = 0.164. **a**, **c** Focal spot profiles and normalized intensity profiles for various wavelengths. **b**, **d** Images of element 5 and 6 in group 5 on the 1951 United States Air Force resolution target formed by the achromatic metalens. Scale bar, 20 μm. **e**, **f** Schematic diagram of a 24-h imaging detection. The right embedded image is the visible and NIR imaging results, respectively. The left embedded image is the schematic diagram of incident light band.

Figure 3 shows further characterization of the asymptotic matching achromatic metalens with NA of 0.164 regarding its focusing and imaging performance. The focal spot profiles of the metalens in the visible and near-infrared range are shown in Fig. 3a, c, respectively. The Strehl ratio from the FDTD simulated and measured beam profiles for the metalenses at each wavelength were also calculated (see Fig. S20). Simulated values are all greater than 0.8, while experimental data exhibit a slight decrease at some wavelengths due to processing errors, indicating that the overall diffraction-limited focusing is realized. In Fig. 3b, d, the imaging performance of a standard United States Air Force resolution target from the metalens is presented under various incoherent illumination lights with a bandwidth of 20 nm. Element 5 and 6 in group 5 of the resolution target were measured to evaluate the achromatic performance of the metalens. High contrast imaging is observed at most wavelengths, demonstrating the function of the metalens in ultra-broadband achromatic imaging. Furthermore, the ultra-broadband achromatic metalens is combined with a general CMOS sensor (response range covering 400~1000 nm) to demonstrate an application scenario of 24 h detection, as illustrated in Fig. 3e, f. In this system, the metalens and CMOS sensor remain unchanged, and the color filter between the metalens and CMOS sensor is switched in different scenarios. During daytime or sufficient light conditions, the color filter allows light with a wavelength less than 650 nm to pass through, achieving broadband white light color imaging (Fig. 3e). In nighttime or insufficient light conditions, the infrared light source is used as supplementary light, and the color filter allows light with a wavelength greater than 650 nm to pass through, achieving broadband infrared imaging. At this time, the CMOS chip works in black-and-white mode (Fig. 3f).

**Fig. 4:**
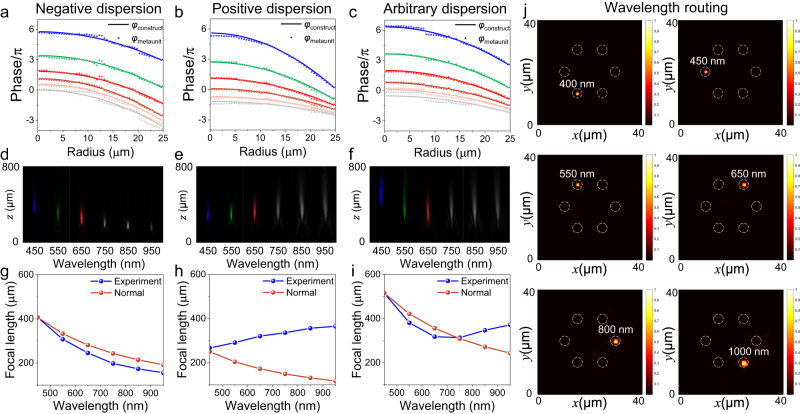
Experimental results of arbitrary dispersion control metalens. **a–c** The matching results of the enhanced negative dispersion, positive dispersion, and arbitrary dispersion manipulation metalenses designed by the asymptotic phase compensation method, respectively, where the solid lines are the constructed wavefront phase profiles at each wavelength, and the scatters are the phase of the matching structural phases. **d–f** Experimental light intensity profiles for the enhanced negative dispersion metalens, positive dispersion metalens and arbitrary dispersion metalens respectively at various incident wavelengths. The unit is nm. **g–i** The blue line is the focal length statistics at all wavelengths, and the crimson line is the normal negative dispersion reference curve. **j** Simulated light intensity distribution of wavelength routing at the focal plane for six wavelengths across 400~1000 nm bandwidth.

To further demonstrate the generalizability of asymptotic phase compensation approaches for ultra-broadband dispersion manipulation, we have designed and fabricated three customized metalenses that can achieve enhanced negative dispersion, positive dispersion, and arbitrary dispersion manipulation. The customized dispersion design is achieved by constructing the wavefront phase at each wavelength for the desired focus, where the asymptotic matching scheme provides great freedom to design each individual wavefront. The metalenses have a diameter of 50 μm and a focal length of 320 μm at wavelength of 650 nm. Figure 4a–c depicts the phase profiles at each wavelength constructed by the asymptotic phase compensation method (solid lines), as well as the matching structural phases (scattered points). It is observed that the phase matching error is small. Furthermore, despite the anomalous dispersion characteristics of the designed metalens, it is noteworthy that the constructed phase relationship among different wavelengths do not exhibit a reversed relationship but maintain the same relationship as the phase dispersion of the meta-unit itself. This phenomenon is attributed to the independent translation of the wavefront of each wavelength, which enables the establishment of a dispersion relationship that is more consistent with the dispersion phase of the meta-unit while achieving the desired focus. Figure 4d–f presents the intensity distribution measurements along the propagation direction for three metalenses in the 400~1000 nm band. All three metalenses exhibit excellent focus at each wavelength, but they have markedly different dispersion properties. Figure 4g–i displays the focal length statistics at all wavelengths, with the blue line serving as the normal negative dispersion reference curve. It is evident that the three designed metalenses have effectively enhanced negative dispersion, positive dispersion, and arbitrary dispersion modulation. Furthermore, our approach supports more complex arbitrary dispersion modulation. For example, we simulated an ultra-broadband wavelength routing that allows customized separation of different wavelengths (six wavelengths in the 400~1000 nm band) with low crosstalk in the focal plane, as shown in Fig. 4j. Such devices have a wide range of applications in areas such as spectral imaging and wavelength division multiplexing.

## Discussion

Our proposed asymptotic phase compensation approach provides a powerful tool for dispersion modulation in ultra-broadband enabling chromatic aberration cancellation in even larger bandwidths. We demonstrate through simulation to achieve an achromatic design with a larger NA or broader bandwidth (400~1500 nm) by increasing the height of the structure to 1500 nm in Fig. S12. This approach still does not break the limit of the maximum dispersion range of the nanostructures, i.e., realizing achromatic metalenses with larger diameters and higher NAs requires a larger range of phase dispersion supported by increasing the refractive index of the material and the height of the meta-units. But this scheme provides a possible idea to break through this limit at discrete wavelengths by wrapping all the phase profiles to $2{{{{{\rm{\pi }}}}}}$ range and then optimizing ${r}_{\lambda }$ for each wavelength (see Section 15 in Supplementary Information). The capability of the method for large area and large NA ($R$ = 500 μm, ${{{{{\rm{NA}}}}}}$ = 0.98, ${{{{{\rm{LPF}}}}}}$ = 48≫1) achromatic metalens design at discrete wavelengths (450, 531 and 633 nm) in the visible is also demonstrated by simulation in Fig. S22. The customized dispersion manipulation capability can be applied in various applications^54–56^ such as color holography^6^, spectral detection^3^, and wave division multiplexing optical communication.

In summary, we proposed a generalized asymptotic phase compensation scheme that better matches the intrinsic phase dispersion response of nanostructures to address the obstacle of large errors in arbitrary dispersion manipulation designs with a linear phase compensation scheme. With this scheme, we have demonstrated ultra-broadband achromatic dielectric metalenses spanning the visible to the near-infrared band from 400 to 1000 nm composed of carefully fabricated high aspect ratio nanostructures. This band covers the response of general CMOS image sensors enabling achromatic imaging in both day and night environments with the same miniaturized optical system. In addition, this scheme provides great freedom to achieve various types of customized dispersion modulation for applications such as color holography and spectral detection. The proposed method improves the theoretical framework of single-layer dispersion-controlled metalenses design, which not only allows for arbitrary dispersion manipulation without bandwidth restrictions but also provides a potential solution to break the maximum phase dispersion limit. The proposed framework is expected to have significant applications in ultra-broadband imaging and spectral detection, among others.

## Methods

### Numerical simulation

The ERIs of 14 kinds of meta-units are simulated by the Lumerical MODE Solutions. The period of meta-units was set as 500 nm. Considering the constraints of experimental conditions and period size, we set the minimum and maximum size constraints of the nanofins to be 50 nm and 450 nm, respectively. Nanostructures with different cross-sectional shapes are shown in Fig. S5. For the simulation, the boundary conditions of the simulation are set to periodic boundary conditions. The refractive index of the TiO_2_ was the measurement result by ellipsometer. We used eigenmodes to analyze and calculate nanostructures with different wavelengths and different cross-sectional shapes, and obtain the equivalent refractive index ${n}_{{{{{\rm{eff}}}}}}$.

### Matching optimization

We will first set a range for ${r}_{\lambda }$, and the algorithm will initially have a random value for ${r}_{\lambda }$. Since the phase dispersion available in the meta-units’ libraries are fixed, all we have to do is to vary the value of ${r}_{\lambda }$ so that the required phase dispersion is covered by the meta-units as much as possible. As we perform each PSO iteration, the required phase dispersion is subtracted from the meta-units phase dispersion and an error value is obtained (see Eq. (S2) in Section 7 of the Supplementary material for details of the error formula). As the number of iterations increases, by the characteristics of the PSO algorithm, ${r}_{\lambda }$ will continue to iterate in a more optimal direction, while the error value decreases. Until we have iterated a certain number of times, the error value is minimized, which is the ideal ${r}_{\lambda }$ value for the metalens.

### Device fabrication

First, a 1000-nm-thick polymethyl methacrylate (PMMA) electron-beam resist layer was spin coated at 2000 rpm on the transparent glass substrate with ITO film layer and baked on a hot plate for 4 min at 180 °C. Then, the sample was exposed by electron-beam lithography (EBL) with a 100-KV voltage and a beam current of 200 pA. Subsequently, the sample was developed in a mixed solution of methyl isobutyl ketone (MIBK) and isopropanol (IPA) (MIBK: IPA = 1:3) for 1 min, and fixed in the IPA for 1 min. Later, we used the atomic layer deposition (ALD) system to fill the exposed area with 230 nm TiO_2_. After this process, a layer of 230 nm TiO_2_ will remain on the top of the entire sample. We etched the TiO_2_ on the top layer by ion beam etching (IBE) in the next process. Then, we used reactive ion etching (RIE) to remove the photoresist with O_2_ gas. Finally, the TiO_2_ structures with a high aspect ratio are obtained.

### Optical characterization

In order to verify the performance of ultra-broadband achromatic metalens and arbitrary dispersion control metalenses, we design two optical setups to characterize the focal length and imaging effect, respectively. Details of the optical experimental setup for characterizing the ultra-broadband achromatic metalens are shown in supplementary information.

### Supplementary information

Supplementary Information

## Data Availability

The data that support the findings of this study have been included in the main text and Supplementary Information. All other relevant data supporting the findings of this study are available from the corresponding authors upon request.

## Code availability

The codes used for the metalens design in this study are available from the corresponding authors upon request.
