# Author Correction: Asymptotic dispersion engineering for ultra-broadband meta-optics

**DOI:** 10.1038/s41467-023-43525-3

**Published:** 2023-11-21

**Authors:** Yueqiang Hu, Yuting Jiang, Yi Zhang, Xing Yang, Xiangnian Ou, Ling Li, Xianghong Kong, Xingsi Liu, Cheng-Wei Qiu, Huigao Duan

**Affiliations:** 1 National Research Center for High-Efficiency Grinding, College of Mechanical and Vehicle Engineering, Hunan University, Changsha, 410082 PR China; 2 Advanced Manufacturing Laboratory of Micro-Nano Optical Devices, Shenzhen Research Institute, Hunan University, Shenzhen, 518000 PR China; 3 Department of Electrical and Computer Engineering, National University of Singapore, Singapore, Singapore; 4 Greater Bay Area Institute for Innovation, Hunan University, Guangzhou, 511300 PR China

**Keywords:** Metamaterials, Nanophotonics and plasmonics

Correction to: *Nature Communications* 10.1038/s41467-023-42268-5, published online 20 October 2023

Supplementary Information Fig. S10 was originally published with an error in the position of the two rows of figures for Fig. S10. Specifically, the positions of the two rows of figures a–c and d–f have to be reversed in Fig. S10. The original article has been corrected, and the Authors have provided the updated Fig. S10 and the Corrected Supplementary Material documents.

Further, except for figure caption of Fig. S10, no other modifications were made in the Supplementary Information.

### Supplementary information

Updated Supplementary Information

