# Supplementary material for: Asymptotic dispersion engineering for ultra-broadband meta-optics
Source: Nat Commun. 2023 Oct 20;14:6649. doi: 10.1038/s41467-023-42268-5 (PMC10589226; doi:10.1038/s41467-023-42268-5)
Supplement: Supplementary file 1 — Supplementary Information [file 41467_2023_42268_MOESM1_ESM.pdf]

Supplementary Information for

Asymptotic Dispersion Engineering for Ultra-Broadband Meta-Optics

Yueqiang Hu[1,2], Yuting Jiang[1], Yi Zhang[1], Xing Yang[1], Xiangnian Ou[1], Ling Li[1], Xianghong Kong[3], Xingsi Liu[3], Cheng-Wei Qiu[3,*] and Huigao Duan[1,2,4,*]

[1]National Research Center for High-Efficiency Grinding, College of Mechanical and Vehicle Engineering, Hunan University, Changsha 410082, P.R. China

[2]Advanced Manufacturing Laboratory of Micro-Nano Optical Devices, Shenzhen Research Institute, Hunan University, Shenzhen, 518000, P.R. China

[3]Department of Electrical and Computer Engineering, National University of Singapore, Singapore, Singapore

[4]Greater Bay Area Institute for Innovation, Hunan University, Guangzhou 511300, P.R. China

[*]Corresponding authors. Email: chengwei.qiu@nus.edu.sg, duanhg@hnu.edu.cn

Section 1: The linear phase compensation method.

As shown in Figure S1a, for the conventional lens wavefront, d_1 is intuitively designed to construct a phase distribution of $\varphi_1 = -\frac{2\pi}{\lambda}\left(\sqrt{r^2+f^2}-f\right)$, where f is the focal length. The dispersion relationship is shown in the embedded figure where the wavelength of the red line is the largest. For a certain position of the lens, the phase is negatively correlated with the wavenumber as shown in Figure S1b. Therefore, it is clear from Eq. (2) that phase compensation cannot be achieved with the meta-units. The scheme commonly used in the previous studies is shown in Figure S1c, where the wavefront is expanded outward to the reference position r_0 and the phase distribution is obtained as $\varphi_{\text{linear}} = -\frac{2\pi}{\lambda}\left(\sqrt{r^2+f^2}-\sqrt{r_0^2+f^2}\right)$. The dispersion relationship in the embedded figure shows that the phase profiles are translated upward and intersected at position r_0. In this way, the phase dispersion sign is flipped at positions smaller than r_0 as shown in Figure S1c, providing the possibility of phase compensation by meta-units. However, since r_0 is a constant resulting in a linear relationship between the constructed phase and the wavenumber, the chromatic aberration can be eliminated only in a narrow bandwidth with an approximate linear fit with the meta-units' dispersion (Figure S1d). It will introduce large error for wider bandwidth and the meta-unit's library selection is limited.

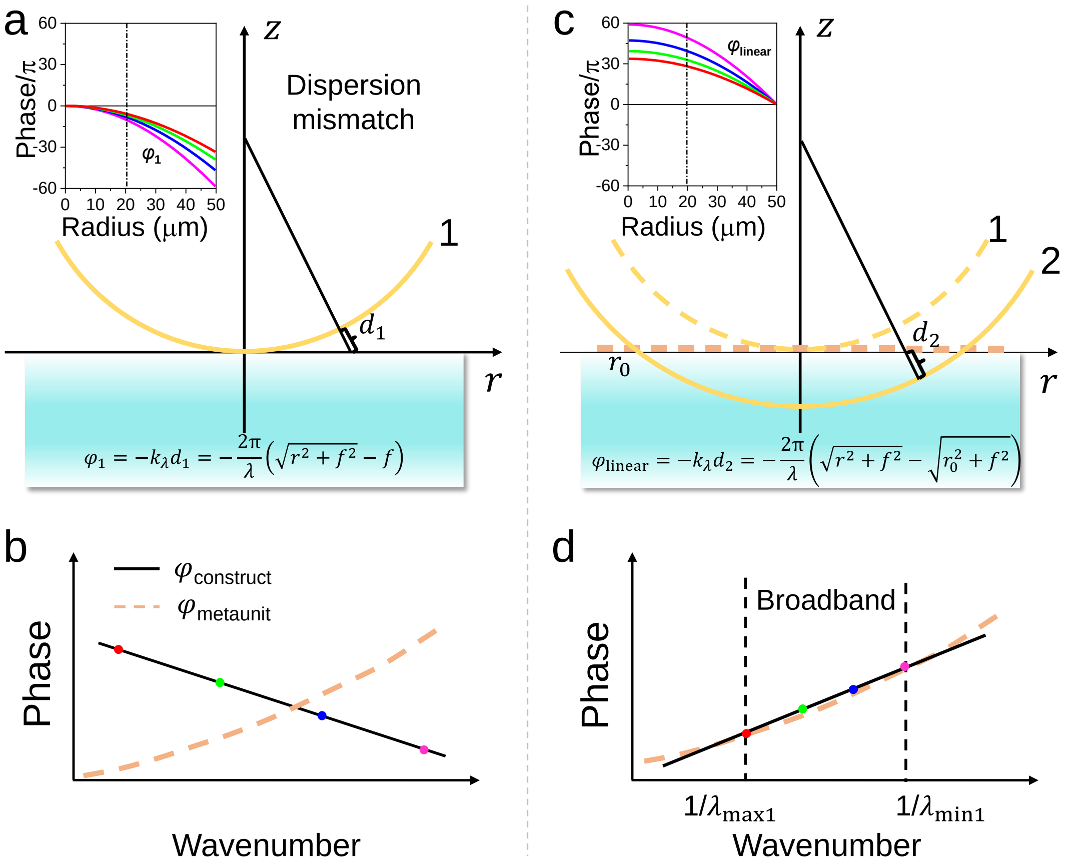

Fig. S1 Schematic of the linear phase compensation method. a, c The schematic wavefronts of a conventional lens and linear phase compensation method. The left embedded figure is the phase profiles of different wavelengths which are getting smaller from red to purple. **b, d** The comparison between the intrinsic phase dispersion of the meta-unit and constructed phase dispersion of traditional lens and linear phase compensation method respectively.

Section 2: Theoretical derivation of wavenumber and effective refractive index.

The formula S1 expresses the relationship between the wavenumber and the effective refractive index (ERI) in the one-dimensional equivalent medium theory.

$$n_{\mathrm{eff}} = \left(\frac{1-F}{n_{\mathrm{air}}^2} + \frac{F}{n_{\mathrm{TiO_2}}^2}\right)^{-1/2} \tag{S1}$$

where F is the duty cycle. n_{eff} is the effective refractive index. n_{air} is the refractive index of air. $n_{\mathrm{Tio_2}}$ is the experimentally measured refractive index of titanium dioxide (TiO_2). Figure S2 shows the relationship between the wavenumber and the effective refractive index derived from the formula S1 when F=0.5.

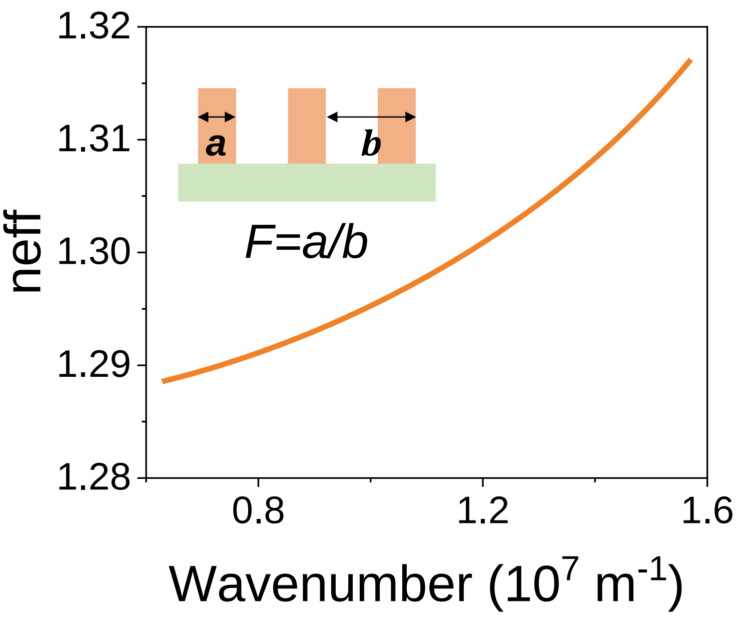

Fig. S2 The relationship between wavenumber and effective refractive index is derived from the one-dimensional equivalent medium theory.

Section 3: Optical constant of TiO_2.

Figure S3 shows the experimentally measured *n* and *k* values of TiO_2 in the 210 nm-1650 nm band.

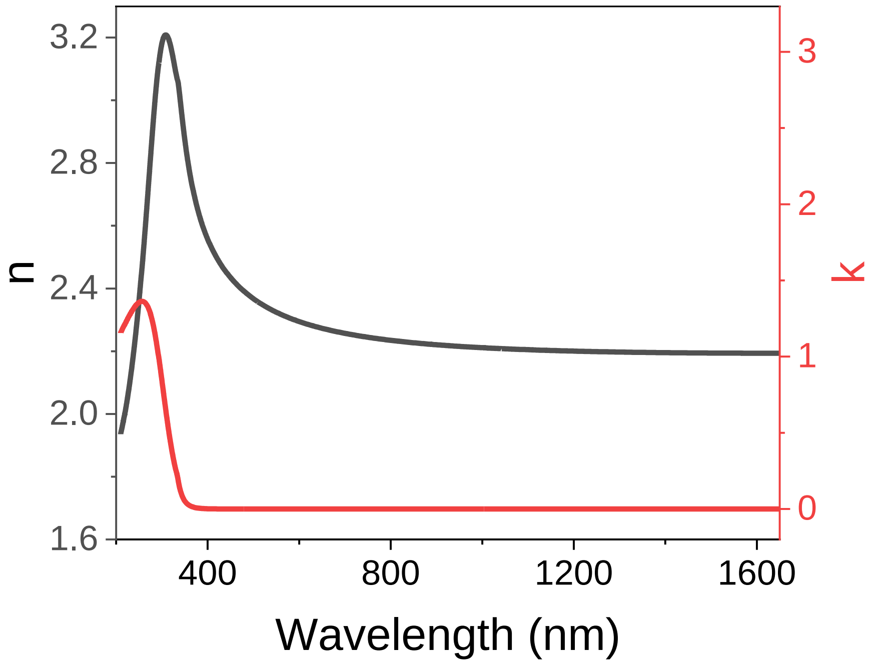

Fig. S3 The optical constant of TiO_2.

Section 4: The intrinsic phase dispersion response of nanostructures.

The relationship between the wavenumber and phase of four meta-units is shown in Figure S4, which proves that the nanostructures have nonlinear phase dispersion.

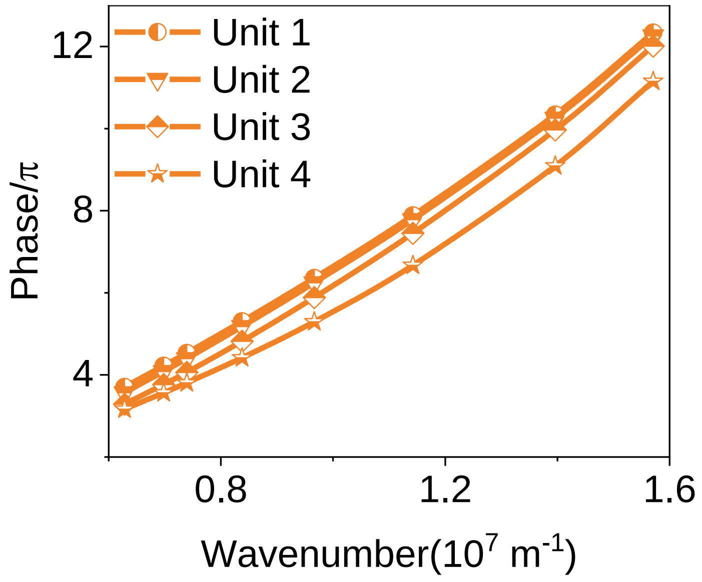

Fig. S4 The intrinsic phase dispersion response of nanostructures.

Section 5: Meta-units' libraries.

First, the ERIs of 14 kinds of meta-units are simulated by the Lumerical MODE Solutions. The period of meta-units was set as 500 nm. Considering the constraints of experimental conditions and period size, we set the minimum and maximum size constraints of the nanostructures to be 50 nm and 450 nm, respectively. Six nanostructures with different cross-sectional shapes are shown in Figure S5. For the simulation, the boundary conditions of the simulation was set to periodic boundary conditions. The refractive index of the TiO_2 was the measurement result by ellipsometer. We used eigenmodes to analyze and calculate nanostructures with different wavelengths and different cross-sectional shapes, and obtain the equivalent refractive index n_{eff}.

Then we calculated the phase at different wavelengths (400 nm, 450 nm, 550 nm, 650 nm, 750 nm, 850 nm, 900 nm, 1000 nm) when the height is 600 nm according to formula 1. We calculated the phase φ_0, at the largest wavelength (λ=1000 nm) and dispersion, $\Delta\varphi = \varphi - \varphi_0$, for the chosen $\Delta\varphi$ (i.e., λ=400~1000 nm) of each meta-units. The calculation results is shown in Figure S6. In the process of matching the structure, we found that it is difficult to achieve ultra-broadband achromatic with these meta-units libraries. In order to obtain better matching results, we changed the structure height to 1000 nm to obtain greater phase compensation. The calculation results is shown in Figure S7.

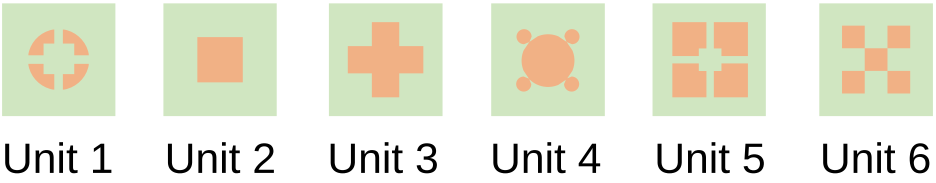

Fig. S5 Schematic of six nanostructures with different cross-sectional shapes.

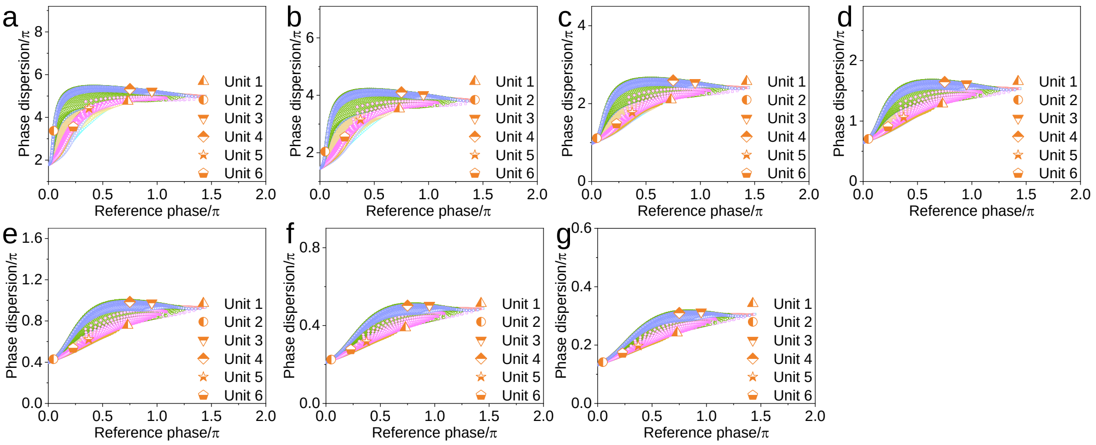

Fig. S6 Meta- units' libraries, the height is 600nm. a λ=400 nm. **b** λ=450 nm. **c** λ=550 nm. **d** λ=650 nm. **e** λ=750 nm. **f** λ=850 nm. **g** λ=900 nm.

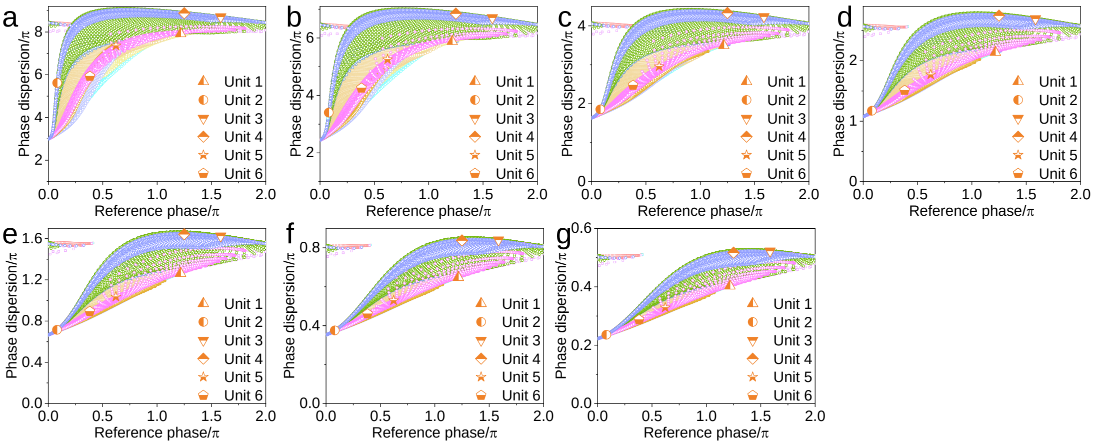

Fig. S7 Meta-units' libraries, the height is 1000nm. a λ=400 nm. **b** λ=450 nm. **c** λ=550 nm. **d** λ=650 nm. **e** λ=750 nm. **f** λ=850 nm. **g** λ=900 nm.

Section 6: The transmission of six meta-units.

Figure S8 shows the transmission of six meta-units, it can be seen from the figure that the transmission of all meta-units is high. Figure S9 shows the transmission simulation of the metalens (NA=0.164). Therefore, the achromatic metalens designed in this work can have high focusing efficiency.

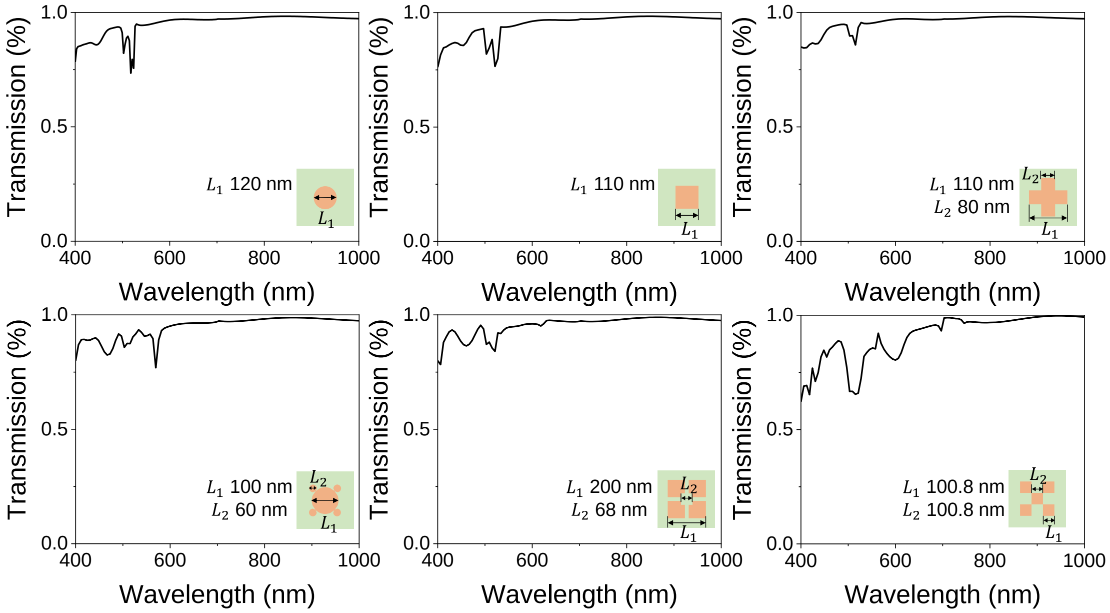

Fig. S8 The simulated transmission of six meta-units.

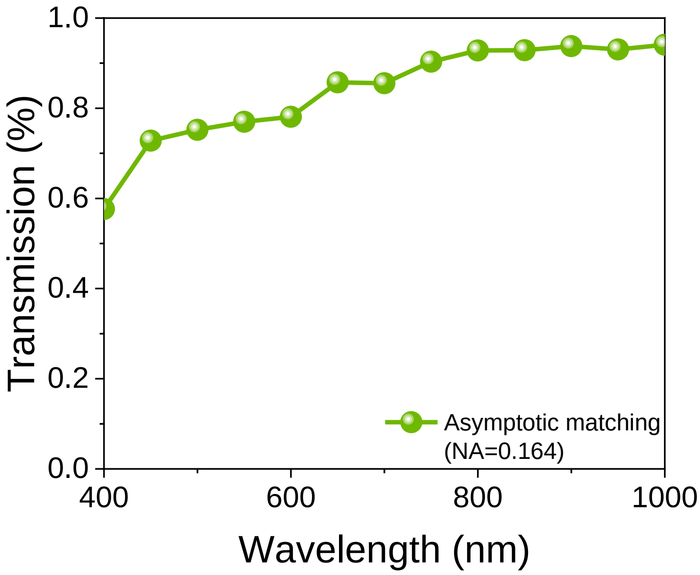

Fig. S9 The simulated transmission of the metalens (NA=0.164).

Section 7: Matching results of ultra-broadband achromatic metalenses with different numerical apertures (NA).

First, we designed ultra-broadband achromatic metalenses with different NAs (0.164, 0.243) in the 400 nm to 1000 nm band by linear dispersive phase compensation methods. The first step is to calculate the expected phase $\varphi_{\mathrm{construct\_0}}$, at the largest wavelength (λ=1000 nm) and expected dispersion, $\Delta\varphi_{\mathrm{construct}} = \varphi_{\mathrm{construct}} - \varphi_{\mathrm{construct\_0}}$,, for the chosen $\Delta\varphi_{\mathrm{construct}}$ (i.e., λ=400~1000 nm) of each meta-units. Then we matched the structures at different r_0, and chosen the r_0 with the smallest error. The error here can be expressed by formula S2, where $\Delta\varphi_{\mathrm{construct}}$ is the expected dispersion, $\Delta\varphi_{\mathrm{metaunit}}$ is the dispersion of the chosen meta-units. r_{max} is the farthest position of the meta-unit on the layout, n_λ is the different wavelength.

$$Error = \sqrt{\sum_{i=1}^{n_\lambda} \sum_{r=1}^{r_{\mathrm{max}}} (\Delta\varphi_{\mathrm{construct}} - \Delta\varphi_{\mathrm{metaunit}})^2} \qquad \text{(S2)}$$

Figure S10a-S10c shows the matching results of the linear dispersion phase compensation method. In order to obtain a smaller matching error, we have proposed an asymptotic dispersion phase compensation method (r_λ is different at different wavelength) in the design of ultra-broadband achromatic metalens. Figure S10d-S10f shows the matching results of the asymptotic dispersion phase compensation method.

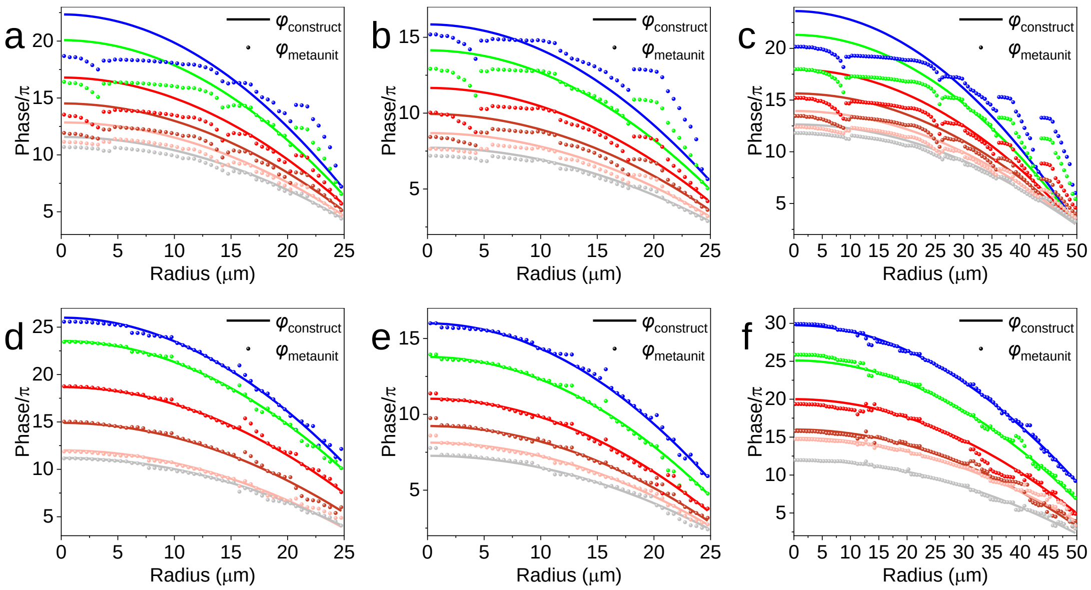

Fig. S10 The linear and asymptotic dispersion phase compensation method matching results with different NA. a-c The linear dispersion phase compensation method matching results. (a. NA=0.243, R=25 μm. b. NA=0.164, R=25 μm. c. NA=0.164, R=50 μm.). **d-f** The asymptotic dispersion phase compensation method matching results. (d. NA=0.243, R=25 μm. e. NA=0.164, R=25 μm. f. NA=0.164, R=50 μm.).

Section 8: The simulation results of the metalenses with different NAs and bandwidths.

In this section we show the simulation results of several metalenses matched by linear and asymptotic compensation approach. Figure S11-S13 shows the simulated intensity distribution along the propagation direction of the metalenses with NA=0.243, R=25 μm, NA=0.164, R=25 μm, NA=0.164, R=50 μm, respectively. In the simulation results of each figure, with the same NA, radius, and matching method, the bandwidth is 350nm, 600nm, and 1100nm, respectively. For the matching of metalenses in the 400~1500 nm band, a large phase compensation is required, so we have increased the height of the nano-structures to 1500 nm.

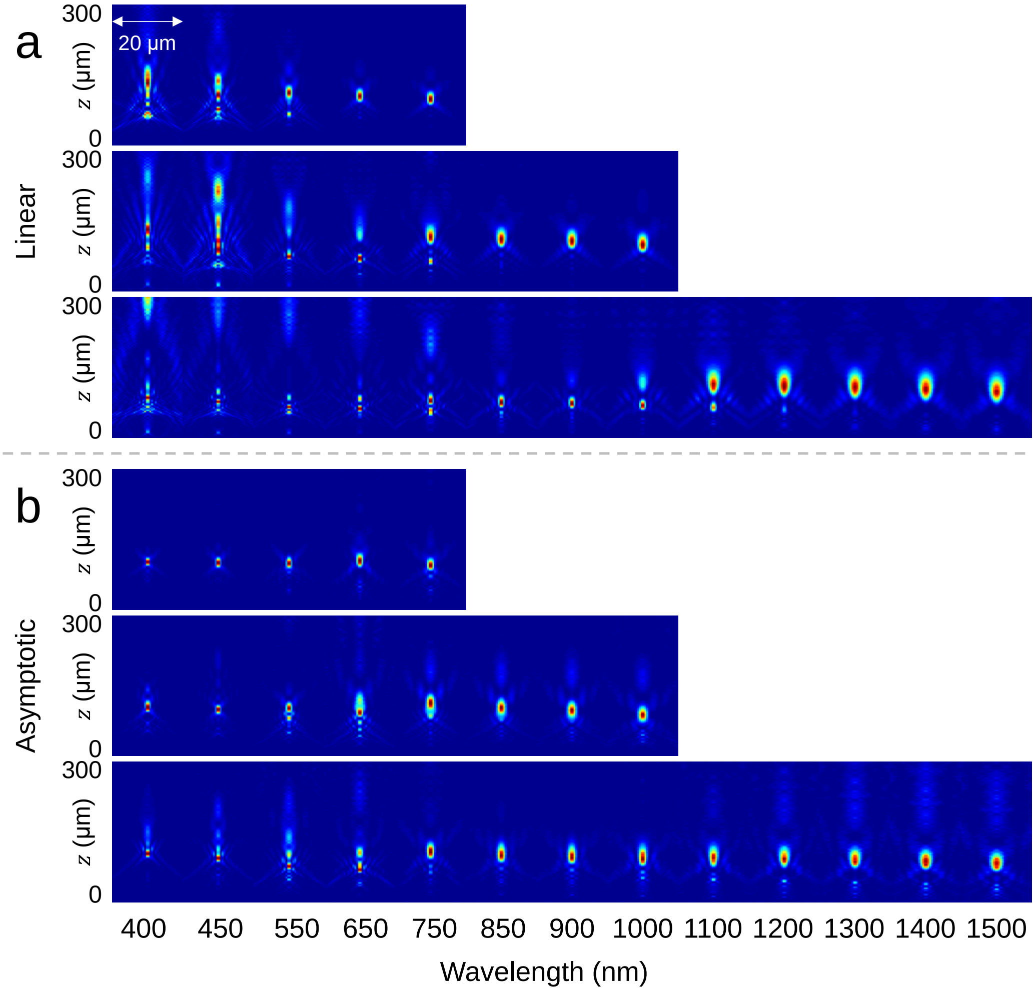

Fig. S11 The simulated intensity distribution along the propagation direction of the metalenses with NA=0.243, R=25 µm. a The simulation results of the linear matched. The bandwidths from top to bottom are 350nm, 600nm and 1100nm, respectively. **b** The simulation results of the asymptotic matched. The bandwidths from top to bottom are 350nm, 600nm and 1100nm, respectively.

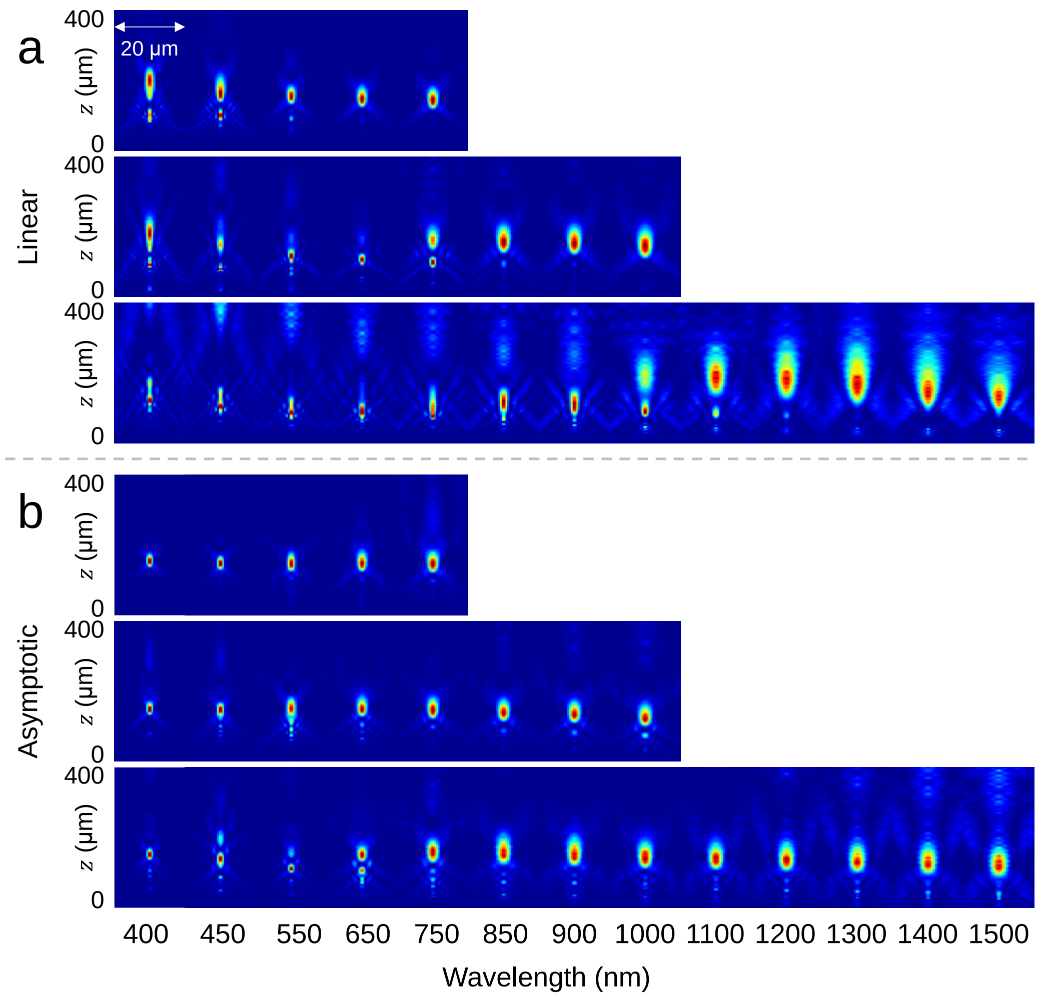

Fig. S12 The simulated intensity distribution along the propagation direction of the metalenses with NA=0.164, R=25 µm. a The simulation results of the linear matched. The bandwidths from top to bottom are 350nm, 600nm and 1100nm, respectively. **b** The simulation results of the asymptotic matched. The bandwidths from top to bottom are 350nm, 600nm and 1100nm, respectively.

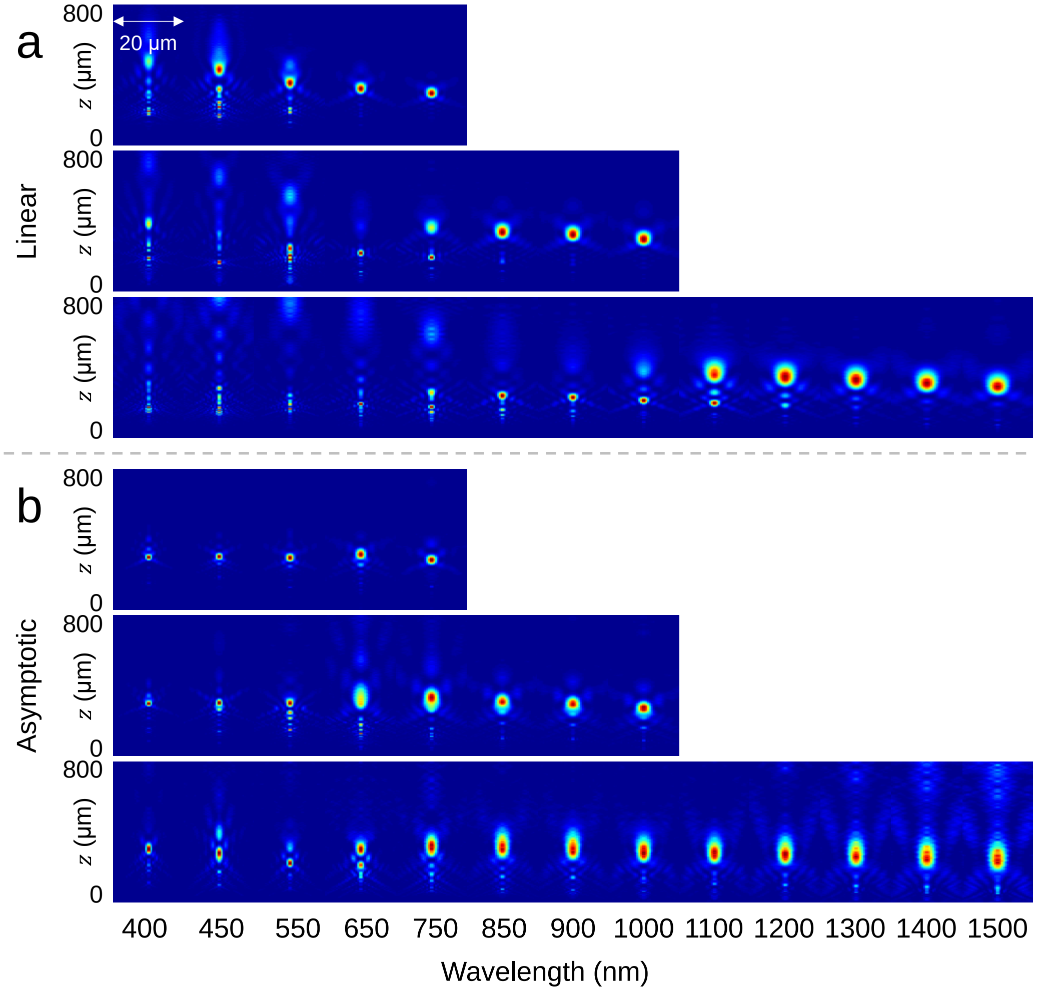

Fig. S13 The simulated intensity distribution along the propagation direction of the metalenses with NA=0.164, R=50 μm. **a** The simulation results of the linear matched. The bandwidths from top to bottom are 350nm, 600nm and 1100nm, respectively. **b** The simulation results of the asymptotic matched. The bandwidths from top to bottom are 350nm, 600nm and 1100nm, respectively.

Section 9: Device fabrication.

The fabrication process of the device is shown in Figure S14, the details are described in the methods section.

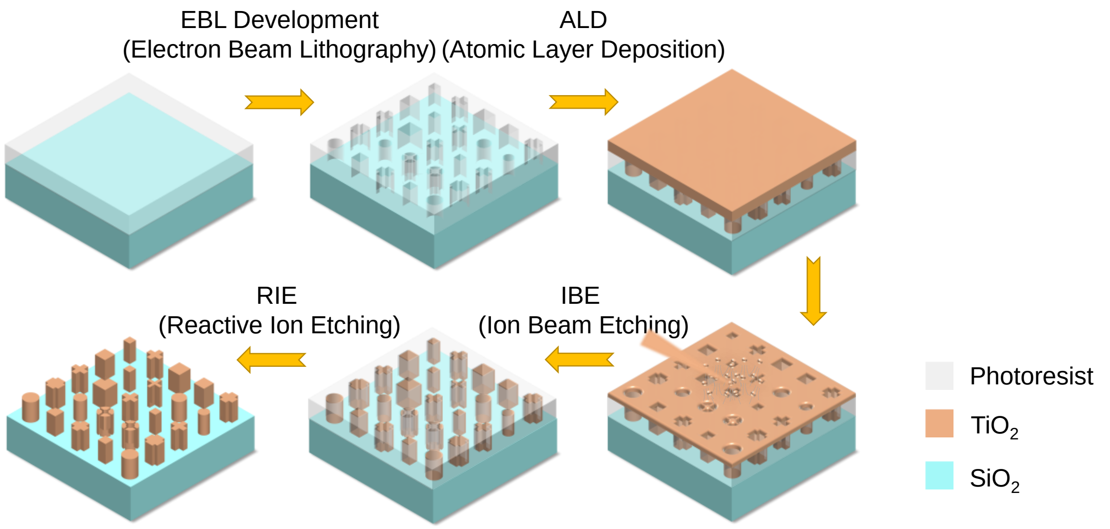

Fig. S14 The fabrication process of TiO_2 structure based on the conformal filling process.

Section 10: Optical characterization.

In order to verify the performance of ultra-broadband achromatic and arbitrary dispersion controlled metalenses, we designed two optical setups to characterize the focal length and imaging effect, respectively.

The optical setup used to verify the ultra-broadband achromatic metalens and the arbitrary dispersion control metalenses is shown in Figure S15. The laser beam is emitted by a supercontinuum laser source, and we can obtain the target wavelength after the laser beam passes through the color filter. The laser beam is imaged on the CCD through the metalens and the objective lens. Finally, the translation stage moved from the position where the sample surface is imaged, and the camera acquired an imaging pattern every 1 μm until it stops at 800 μm from the starting position.

In the imaging experiment, we replaced the super continuum laser source with a halogen lamp. We placed the resolution target in front of the ultra-broadband achromatic metalens and obtained the imaging result at the focal point of the lens. Finally, we judged the imaging quality of the ultra-broadband achromatic metalens by comparing images of different wavelengths.

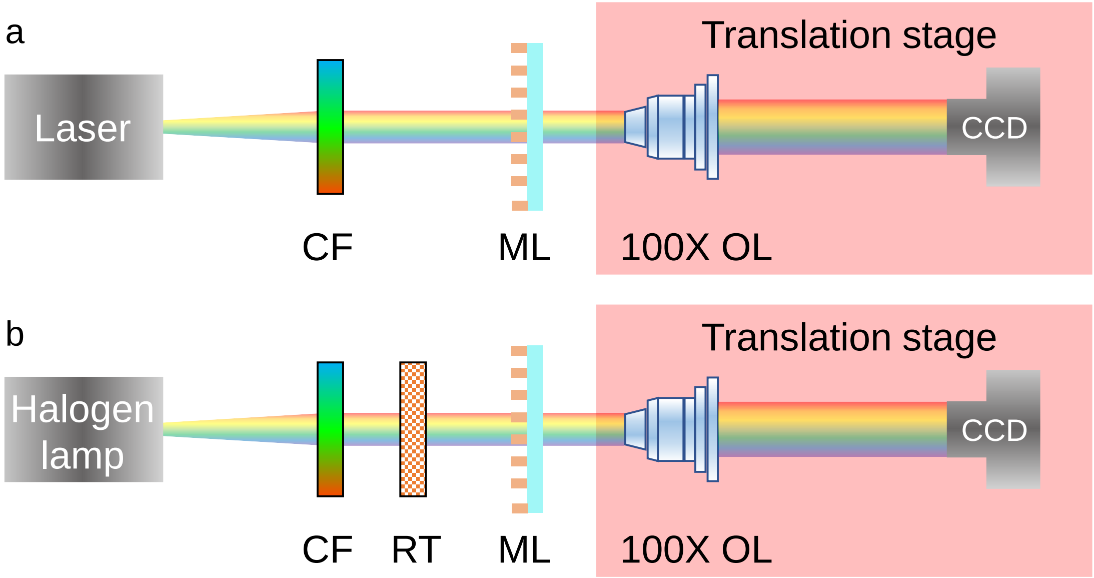

Fig. S15 The optical characterization system. a Schematic diagram of optical setup for verifying the performance of fabricated ultra-broadband achromatic metalens and arbitrary dispersion control metalenses. CF: color filter. ML: metalenses. OL: objective lens. **b** Schematic diagram of optical setup for imaging with broadband achromatic metalenses. RT: resolution target.

Section 11: Focusing efficiency calculation method.

Figure S16 shows the measured focusing efficiency of the asymptotic matching metalens (NA=0.164), where the focusing efficiency is calculated by the intensity within three times the full width half height (FWHM) at the focus plane divided by the intensity of incident light within the range of the metalens. It can be seen that the designed metalens achieves achromatic focusing while also having high focusing efficiency in the operation band. Figure S17 shows the performance of the single-layer achromatic metalens achieved in this work compared to previous ones in experiments.

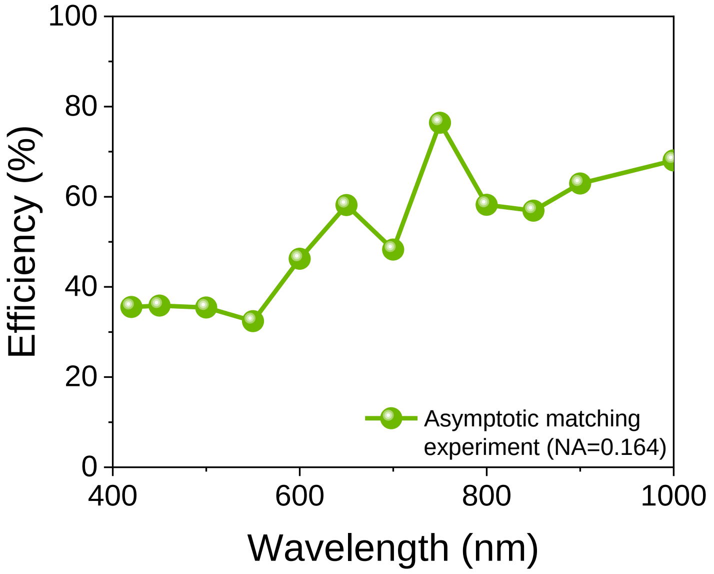

Fig. S16 The measured focusing efficiency of the asymptotic matching metalens (NA=0.164).

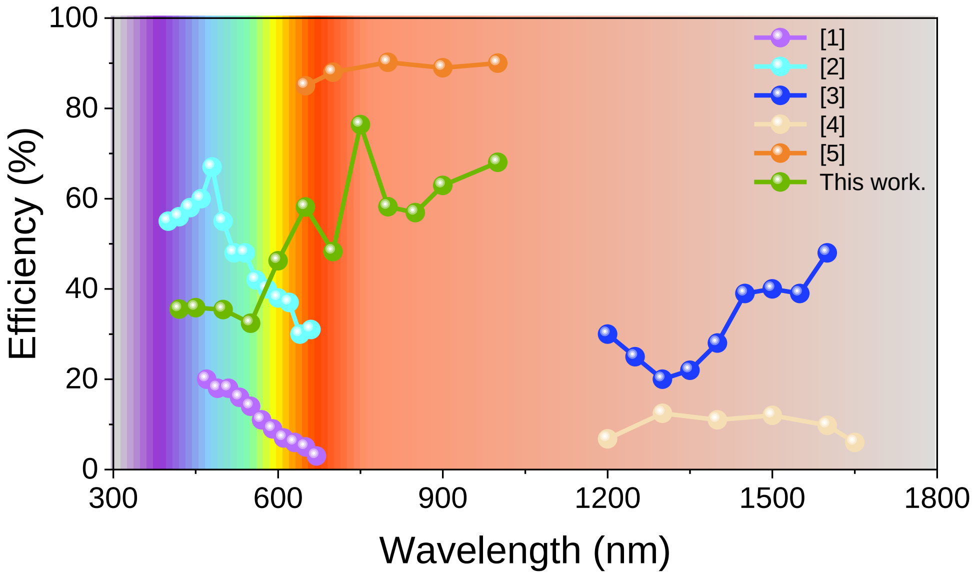

Fig. S17 The efficiencies of broadband achromatic metalenses in different experimental works.

Section 12: The effects of processing errors.

In fact, some errors will inevitably be introduced in every step of sample preparation. Therefore, for the processing plan used in this article, we had selected two locations where errors are most likely to occur and performed corresponding simulations. First, we simulated the overexposure caused by the large dose in the electron beam exposure process. The left side of Figure S18 is the simulation result with normal structure size, and the result on the right is the simulation result after all the structure sizes are increased by 40 nm. We also simulated the loss of the structure height during the ion beam etching process. The left side of Figure S19 is the simulation result when the height of the nanostructure is 1000 nm, and the right side is the simulation result when the structure height is reduced to 800 nm.

In the process of comparing these two sets of simulation results, it can be found that whether the size of the nanostructure increases or the height decreases, the focal length will have a larger deviation at a short wavelength, while the impact will be less at a long wavelength. Part of the reason is that nanostructures need to provide greater phase compensation at shorter wavelengths, and the deviation of the structure will cause a bigger phase change, which will cause a change in the focal length.

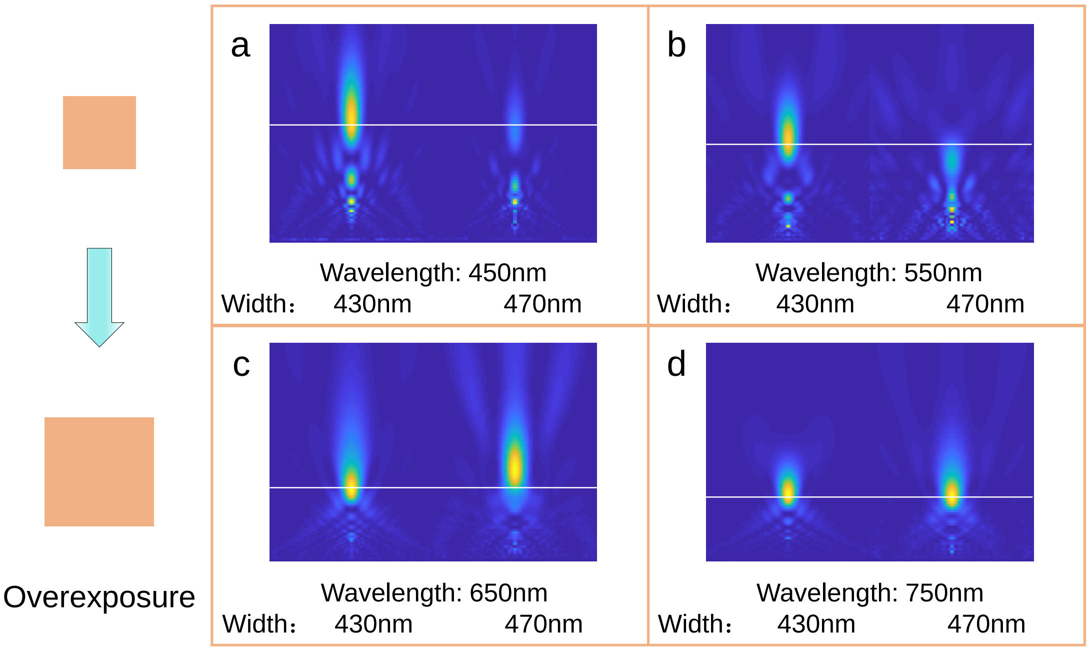

Fig. S18 Simulation of the impact of overexposure on the focusing effect. a-d Comparison of simulation results when λ =450 nm. 550 nm. 650 nm. 750 nm.

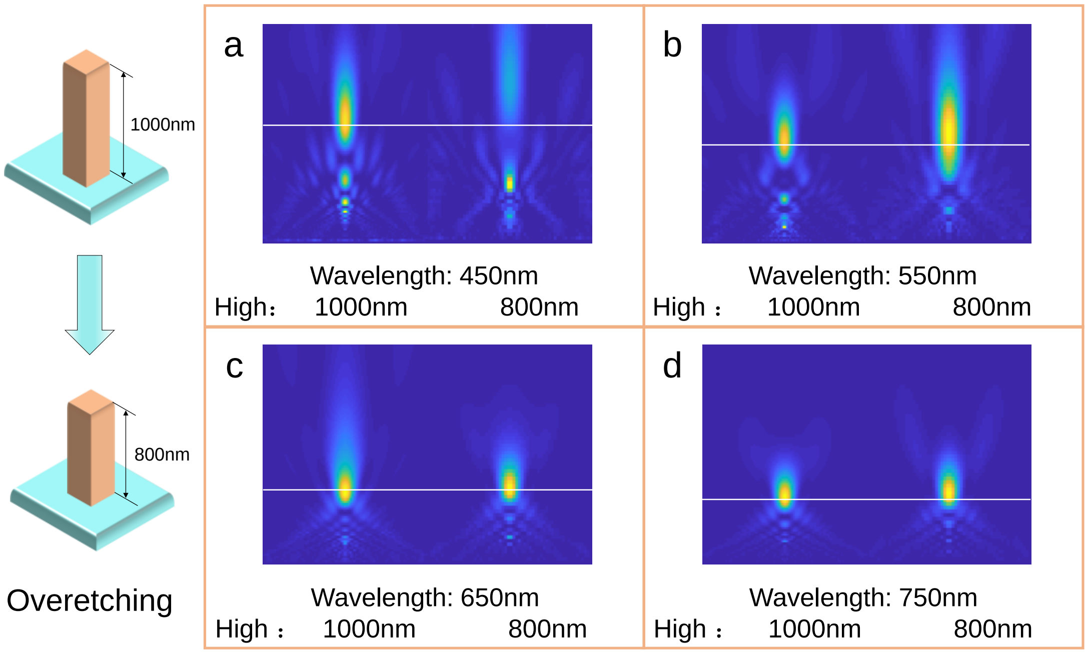

Fig. S19 Simulation of the impact of overetching on the focusing effect. a-d Comparison of simulation results when λ =450 nm. 550 nm. 650 nm. 750 nm.

Section 13: Comparison of data for single-layer achromatic metalenses.

By comparison with the previous works of single-layer broadband achromatic metalenses in Table S1, in both simulations and experiments, we stay well ahead in terms of bandwidth and *LPF* while maintaining comparable lens size and NA.

Table S1. The comparison of single-layer broadband achromatic metalenses

	$\Delta\lambda$ (nm)	λ_{min} (nm)	H (nm)	R (μm)	NA	LPF $\frac{R\cdot NA\cdot\pi\cdot(1/\lambda_{min}-1/\lambda_{max})}{\Delta\varphi_{max}}$	
[1]	200	470	600	12.85	0.2	0.44	Experiment
[2]	260	400	800	25	0.106	0.42	
[3]	450	1200	1400	50	0.24	0.43	
[5]	350	650	1500	12.5	0.1	0.11	
**This work**	**600**	**400**	**1000**	**25**	**0.1644**	**0.78**	
[6]	950	450	1500	23	0.107	0.52	Simulation
[7]	250	1400	1400	12.3	0.24	0.06	
[8]	200	500	800	8.925	0.16	0.18	
[9]	350	450	600	5	0.2879	0.36	
[10]	100	300	1200	4	0.384	0.14	
[11]	340	400	400	9.9	0.165	0.59	
**This work**	**600**	**400**	**1500**	**25**	**0.2425**	**0.77**	
**This work**	**1100**	**400**	**1500**	**25**	**0.1644**	**0.63**	
**This work**	**450 nm/531 nm/633 nm**		**1000**	**500**	**0.98**	**48**	

Section 14: Calculating strehl ratio.

As a conventional criterion for focusing performance, the Strehl ratio (SR), which is defined as the peak intensity normalized to that of the Airy disk, increases with decreasing WAF_{rms}. As described in article, the WAF_{rms} we calculated of the achromatic metalenses are very small. According to the Maréchal Criterion, when the *RMS* is less than 0.071, the Strehl ratio is greater than 0.8, resulting in the theoretically diffraction-limited focusing. Our calculated values of the Strehl ratio of the focused spot from the FDTD simulation also confirm the conclusion in Figure 2c. However, due to the existence of errors in the actual fabrication, the final wavefront error will be further enlarged, so the Strehl ratio of the actual fabricated metalens is decreased shown in in Figure S20.

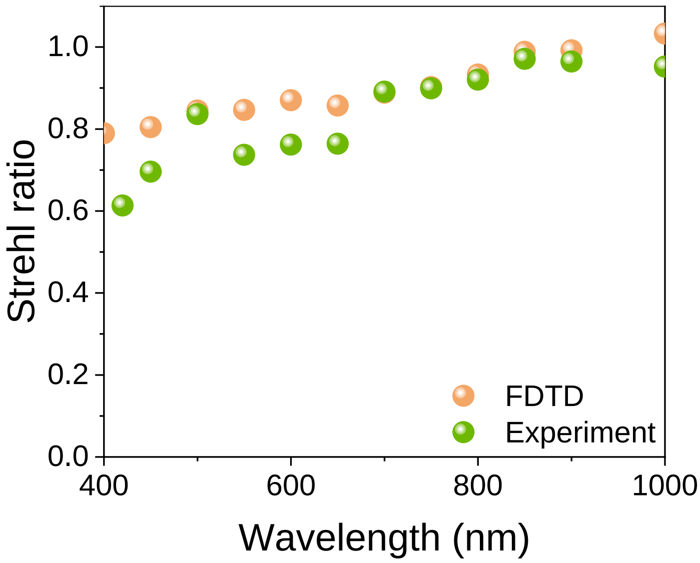

Fig. S20 Simulated and experiment Strehl ratios of metalenses.

Section 15: Design of large area achromatic metalens based on asymptotic dispersive phase compensation.

The design challenge for large area achromatic metalens is the inability to compensate for large dispersion phases using a single structure. Figures S21a and S20b show the phase distribution for three wavelengths with the same NA and different areas. It can be seen that increasing the radius of the metalens from 50μm to 500μm also increases the range of phase compensation required from the nanostructure by a factor of ten to a maximum of 24π, which is well beyond the range of phase compensation that can be provided by the nanostructure. The result of the phase dispersion matching is shown in Figure S21c, which can only satisfy the phase requirements for one wavelength. Accordingly, this scheme folds all phases to the 2π range for matching the structure. Figures S21d and S21e show the matching results of the linear and asymptotic dispersive phase compensation schemes after collapsing the phases, respectively, where the first scheme suffers from a large matching error, while the second scheme achieves a change in the shape and position of the constructed phase curve by modulating r_λ at different wavelengths, and minimize the matching error.

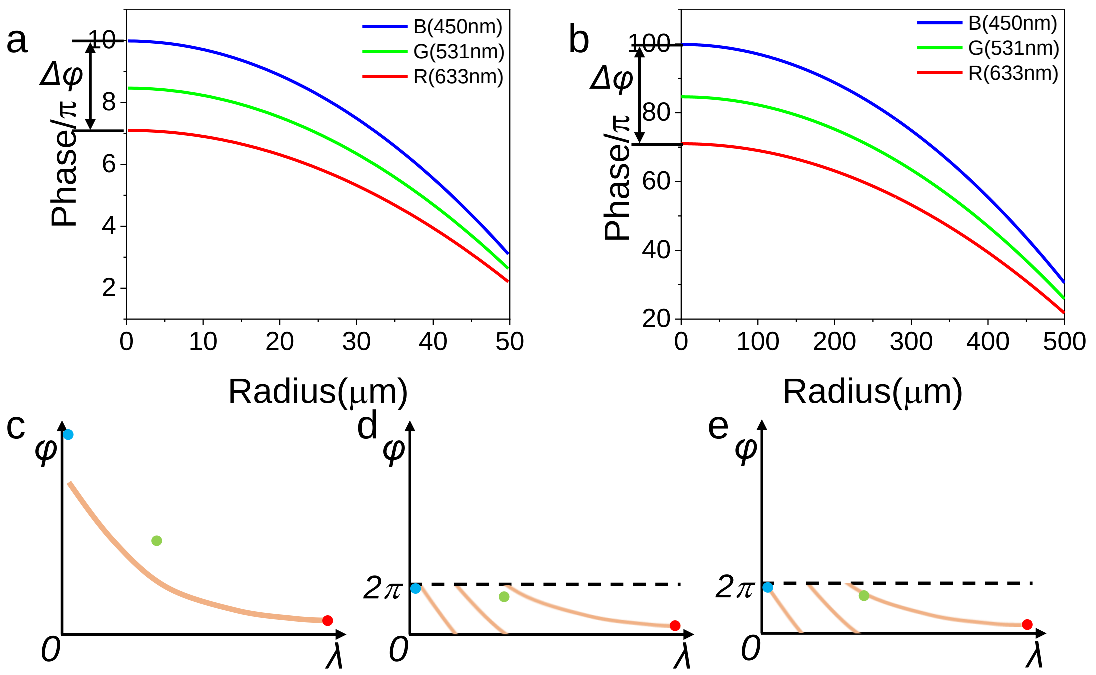

Fig. S21 Schematic diagram of the design principle of a large-area achromatic metalens based on asymptotic dispersion phase compensation. a, b Schematic diagram of the phase distribution of metalens with radii of 50μm and 500μm, respectively. **c** Schematic diagram of phase dispersion before phase folding. **d, e** Schematic diagram of phase dispersion using linear and asymptotic after phase folding, respectively.

Subsequently, we designed an achromatic metalens with R=500μm, f=100μm, NA=0.98 and the working wavelengths are three primary colors R(633nm), G(531nm), and B(450nm) by combining the above scheme and the particle swarm algorithm. Figure S22 shows the results of the scalar diffraction far-field simulation using MATLAB, from which it can be seen that the position of the focal point at 100μm which is in perfect agreement with the designed focal length.

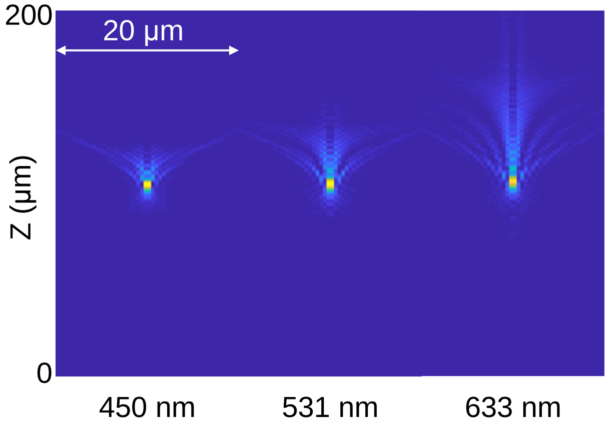

Figure S22 Scalar diffraction simulation of the metalens along the propagation direction (z-axis) with NA=0.98.

Supplementary References

1. Chen, W. T. et al. A broadband achromatic metalens for focusing and imaging in the visible. Nat. Nanotechnol. 13, 220-226 (2018).
2. Wang, S. et al. A broadband achromatic metalens in the visible. Nat. Nanotechnol. 13, 227-232 (2018).
3. Shrestha, S. et al. Broadband achromatic dielectric metalenses. Light Sci Appl. 7, 85 (2018).
4. Wang, S. et al. Broadband achromatic optical metasurface devices. Nat. Commun. 8, 187 (2017).
5. Wang, Y. et al. High-efficiency broadband achromatic metalens for near-IR biological imaging window. Nat. Commun. 12, 5560 (2021).
6. Sun, P. et al. Broadband achromatic polarization insensitive metalens over 950 nm bandwidth in the visible and near-infrared. Chin. Opt. Lett. 20, 13601 (2022).
7. An, X. et al. Broadband achromatic metalens design based on deep neural networks. Opt. Lett. 46, 3881-3884 (2021).
8. Qian Z. et al. Broadband achromatic longitudinal bifocal metalens in the visible range based on a single nanofin unit cell. Optics Express, 30, 11203-11216 (2022).
9. Liu M. et al. Broadband achromatic metalens for linearly polarized light from 450 to 800 nm. Applied Optics, 60, 9525-9529 (2021).
10. Liu M. et al. Polarization independent and broadband achromatic metalens in ultraviolet spectrum. Optics Communications, 497, 127182 (2021).
11. Cheng W. et al. Genetic algorithms designed ultra-broadband achromatic metalens in the visible. Optik, 258, 168868. (2022).
